# Supplementary figures and images for: Simulation study to evaluate when Plasmode simulation is superior to parametric simulation in estimating the mean squared error of the least squares estimator in linear regression
Source: PLoS One. 2024 May 15;19(5):e0299989. doi: 10.1371/journal.pone.0299989 (PMC11095703; doi:10.1371/journal.pone.0299989)

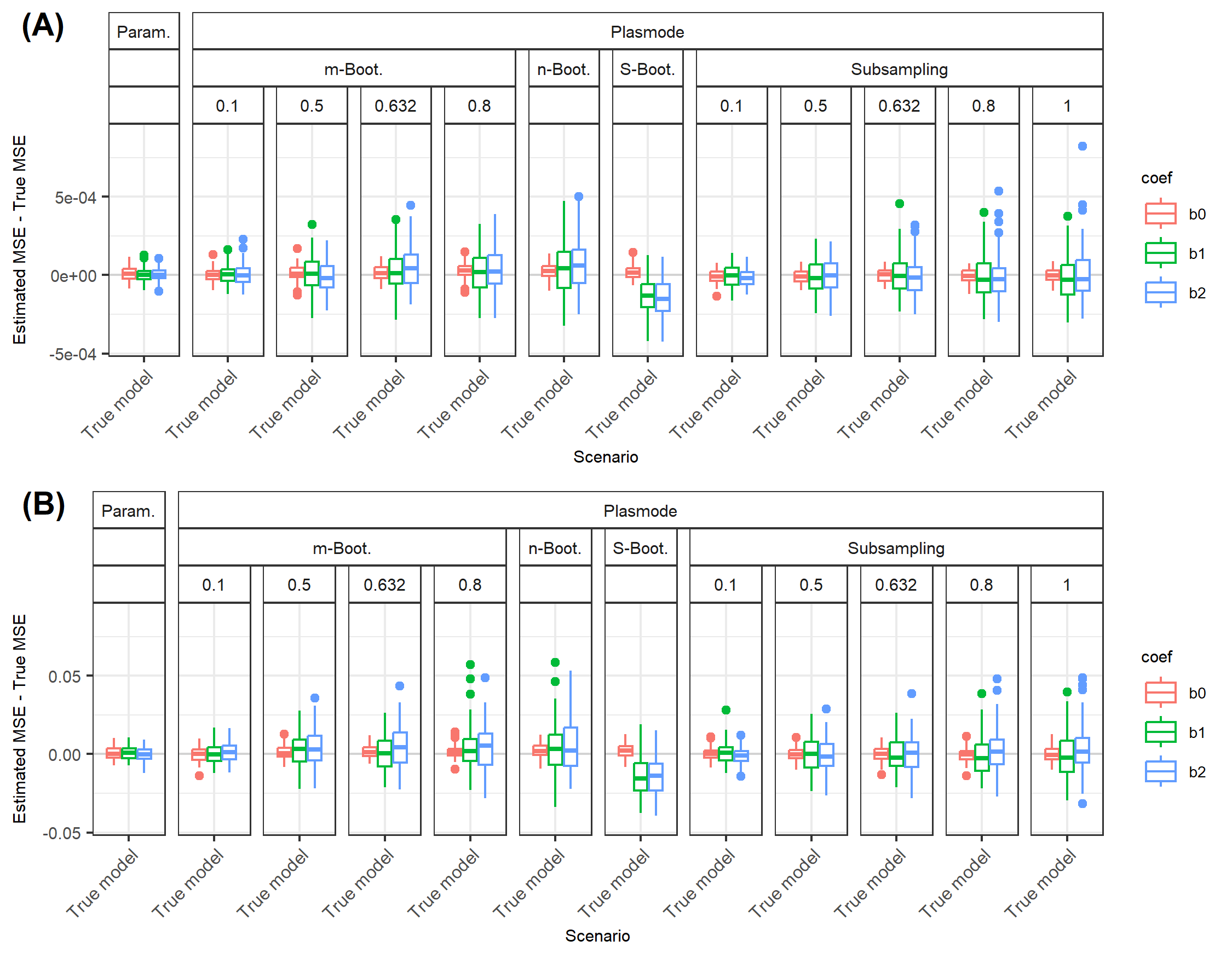

Supplement: S1 Fig — (A) p = 2, n = 100, β = (1, 1, 1)T, σ = 0.3, Cor(Xi, Xj) = 0.2 ∀i ≠ j. (B) p = 2, n = 100, β = (1, 1, 1)T, σ = 3, Cor(Xi, Xj) = 0.2 ∀i ≠ j. (TIF) [file pone.0299989.s001.tif]

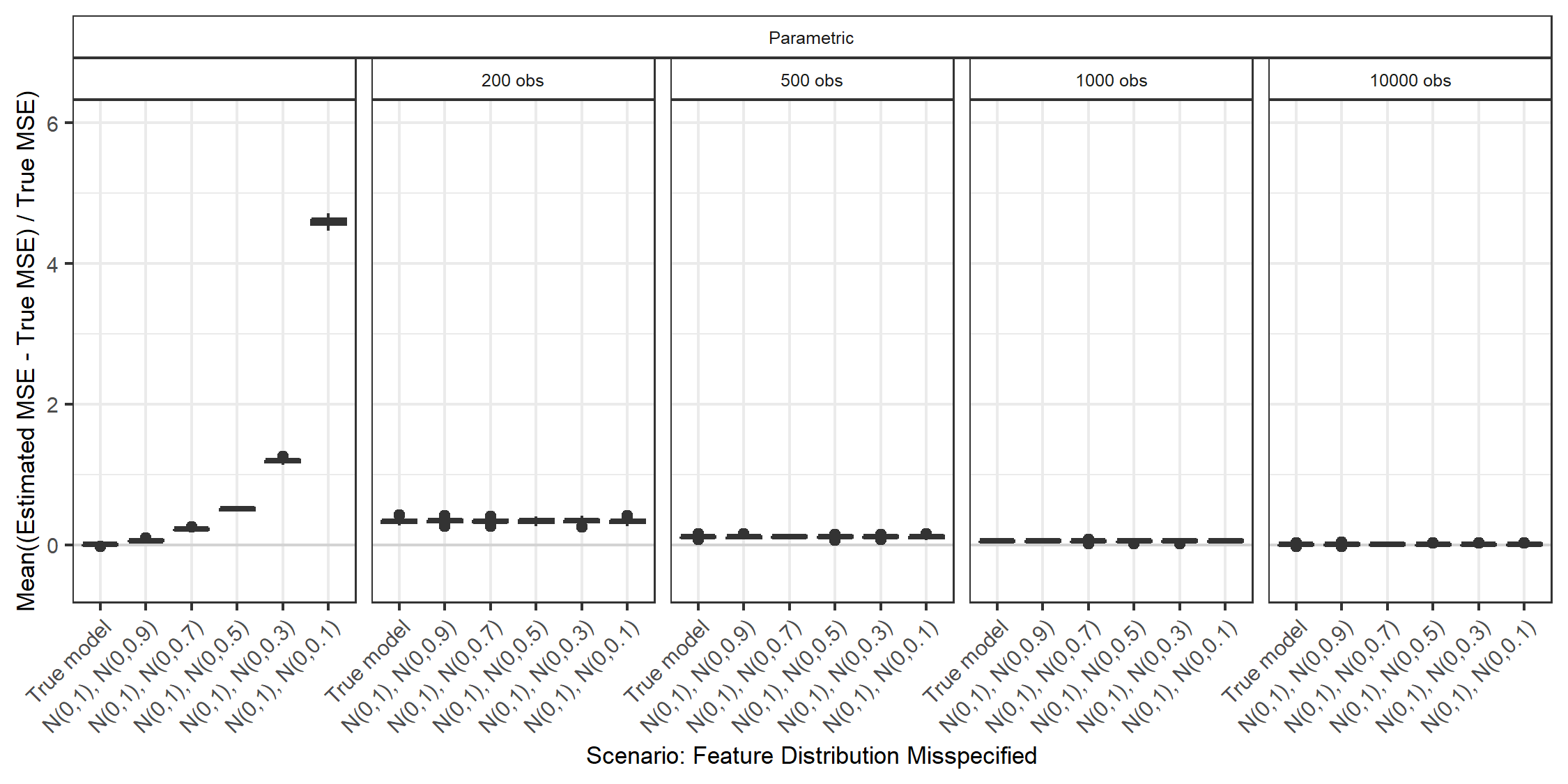

Supplement: S2 Fig — The first facet displays the errors in case the misspecified variances are used in the simulation. The remaining facets display the errors for using a variance that is estimated using datasets of different sizes from the true DGP for parametric simulation instead. (TIF) [file pone.0299989.s002.tif]

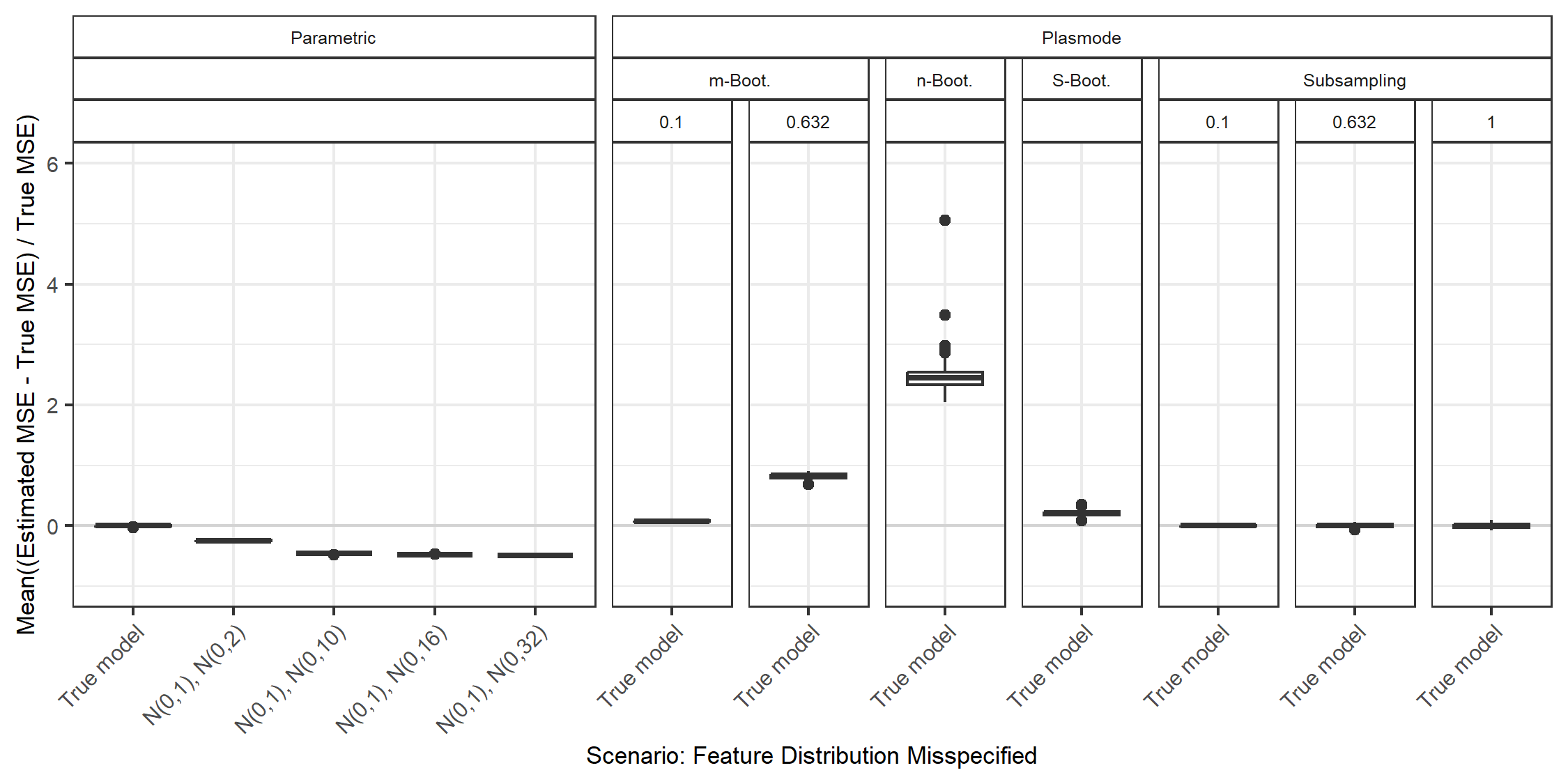

Supplement: S3 Fig — Large outliers for n out of n Bootstrap not displayed. (TIF) [file pone.0299989.s003.tif]

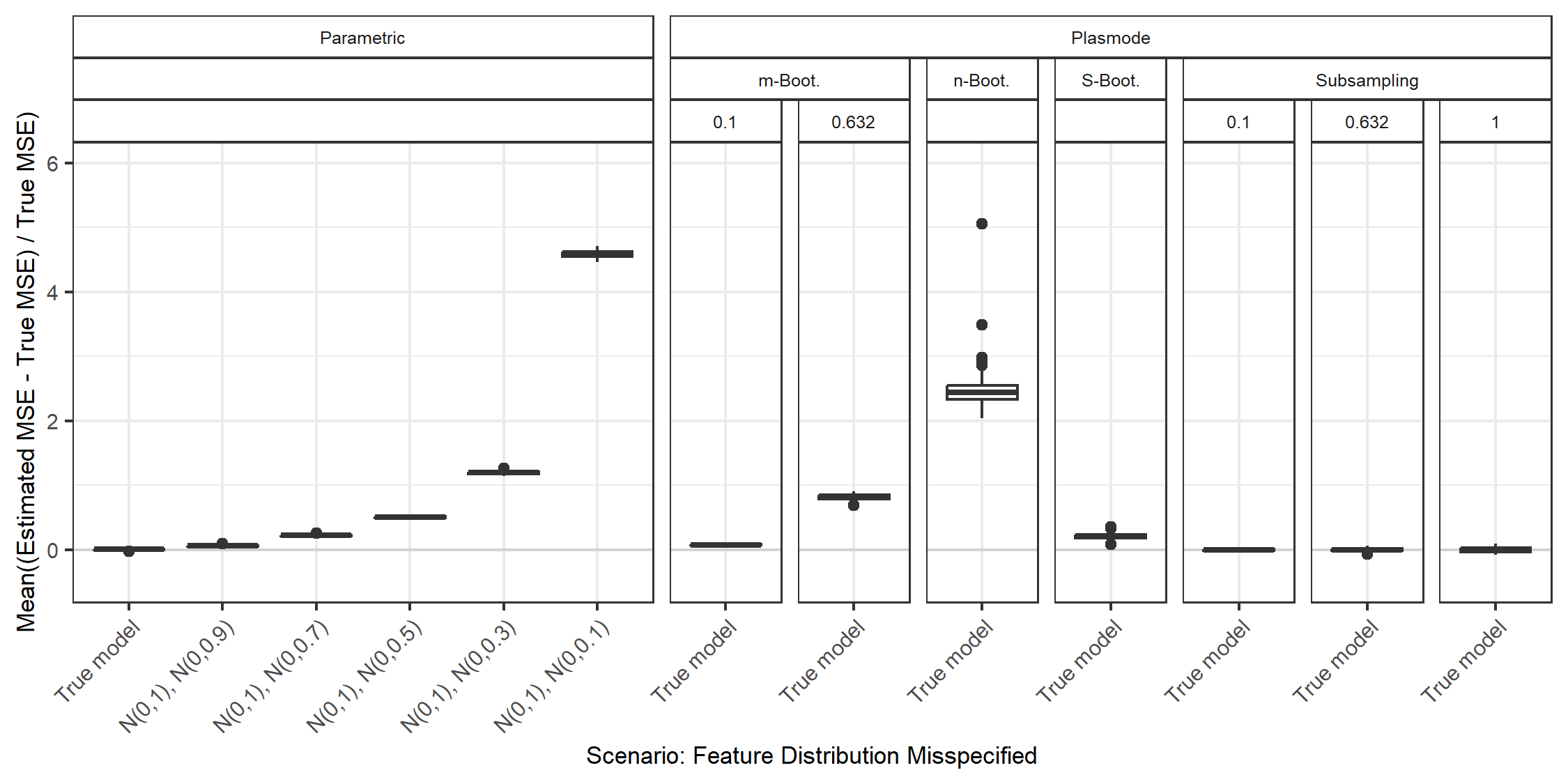

Supplement: S4 Fig — Large outliers for n out of n Bootstrap not displayed. (TIF) [file pone.0299989.s004.tif]
