# Supplementary material for: Simulation study to evaluate when Plasmode simulation is superior to parametric simulation in estimating the mean squared error of the least squares estimator in linear regression
Source: PLoS One. 2024 May 15;19(5):e0299989. doi: 10.1371/journal.pone.0299989 (PMC11095703; doi:10.1371/journal.pone.0299989)
Supplement: S1 Table — (PDF) [file pone.0299989.s005.pdf]

S1 Table to: When is Plasmode simulation superior to parametric simulation when estimating the MSE of the least squares estimator in linear regression?

Marieke Stolte[1*], Nicholas Schreck[2], Alla Slynko[3], Maral Saadati[2], Axel Benner[2], Jörg Rahnenführer[1], Andrea Bommert[1]

1 Department of Statistics, TU Dortmund University, Dortmund, North Rhine-Westphalia, Germany
2 Division of Biostatistics, German Cancer Research Center, Heidelberg, Baden-Wuerttemberg, Germany
3 Department of Statistics and Actuarial Science, University of Waterloo, Waterloo, Ontario, Canada

Table 1. Complete list of deviations from true scenarios

True scenario	Type of deviation	Values
$(p2n100\rho0.2)$	Error sd misspecified	$\sigma \in \{0.1, 0.2, 0.4, 0.5\}$
$(p2n100\rho0.2)$	Correlation misspecified	$\rho \in \{-0.9, -0.8, \ldots, 0, 0.01, \ldots, 0.19, 0.21, 0.22, \ldots, 0.5, 0.6, \ldots, 0.9\}$
$(p2n100\rho0.2)$	Coefficients and correlation misspecified	β_I and $\rho = -0.5$ or β_I and $\rho = 0.5$ or β_{II} and $\rho = -0.5$
$(p2n100\rho0.2)$	Error sd and correlation misspecified	$\sigma = 0.4$ and $\rho = -0.5$ or $\sigma = 0.4$ and $\rho = 0.5$
$(p2n100\rho0.2)$	Expectation of second feature misspecified	$\mu \in \{0.05, 0.1, \ldots, 1, 2, 3\}$
$(p2n100\rho0.2)$	Expectation of both features misspecified	$\mu = 10$
$(p2n100\rho0.2)$	Variance of second feature misspecified	$\sigma^2 \in \{1.05, 1.1, \ldots, 1.5, 2, 3, 5\}$
$(p2n100\rho0.2)$	Variance of both features misspecified	$\sigma^2 = 5$
$(p2n100\rho0.2)$	Mean and variance of both features misspecified	$\mu = 10$, $\sigma^2 = 5$
$(p2n100\rho0.2)$	Distribution of second feature misspecified as Gaussian mixture with $N(0, 10)$	$\alpha \in \{0.01, 0.02, \ldots, 0.05\}$
$(p2n100\rho0.2)$	Distribution of second feature misspecified as Gaussian mixture with $N(3, 1)$	$\alpha \in \{0.01, 0.02, \ldots, 0.05\}$
$(p2n100\rho0.2)$	Distribution of second feature misspecified as log-normal	$logN(0, 1)$
$(p2n100\rho0.2)$	Distribution of second feature misspecified as Bernoulli	$\pi \in \{0.2, 0.35, 0.4, 0.45, 0.5\}$
$(p2n100\rho0.2)$	Error distribution misspecified	$t(10)$, $t(3)$, $\chi^2(1)$, $\chi^2(5)$
$(p2n50\rho0.2)$	Error sd misspecified	$\sigma \in \{0.1, 0.2, 0.4, 0.5\}$
$(p2n50\rho0.2)$	Correlation misspecified	$\rho \in \{-0.9, -0.8, \ldots, 0, 0.01, \ldots, 0.19, 0.21, 0.22, \ldots, 0.4, 0.5, \ldots, 0.9\}$
$(p2n50\rho0.2)$	Coefficients and correlation misspecified	β_I and $\rho = -0.5$ or β_I and $\rho = 0.5$ or β_{II} and $\rho = -0.5$
$(p2n50\rho0.2)$	Error sd and correlation misspecified	$\sigma = 0.4$ and $\rho = -0.5$
$(p2n50\rho0.2)$	Expectation of second feature misspecified	$\mu \in \{0.05, 0.1, \ldots, 1, 2, 3\}$
$(p2n50\rho0.2)$	Expectation of both features misspecified	$\mu = 10$
$(p2n50\rho0.2)$	Variance of second feature misspecified	$\sigma^2 \in \{1.05, 1.1, \ldots, 1.5, 2, 3\}$
$(p2n50\rho0.2)$	Variance of both features misspecified	$\sigma^2 = 5$
$(p2n50\rho0.2)$	Mean and variance of both features misspecified	$\mu = 10$, $\sigma^2 = 5$
$(p2n50\rho0.2)$	Distribution of second feature misspecified as Gaussian mixture with $N(0, 10)$	$\alpha \in \{0.01, 0.02, \ldots, 0.05\}$

Table 1. Complete list of deviations from true scenarios

True scenario	Type of deviation	Values
($p2n50\rho0.2$)	Distribution of second feature misspecified as Gaussian mixture with $N(3,1)$	$\alpha \in \{0.01, 0.02, \ldots, 0.05\}$
($p2n50\rho0.2$)	Distribution of second feature misspecified as log-normal	$logN(0,1)$
($p2n50\rho0.2$)	Distribution of second feature misspecified as Bernoulli	$\pi \in \{0.2, 0.35, 0.4, 0.45, 0.5\}$
($p2n50\rho0.2$)	Error distribution misspecified	$t(10)$, $t(3)$, $\chi^2(1)$, $\chi^2(5)$
($p2n100\rho0.5$)	Error sd misspecified	$\sigma \in \{0.1, 0.2, 0.4, 0.5\}$
($p2n100\rho0.5$)	Correlation misspecified	$\rho \in \{-0.9, -0.8, \ldots, 0.4, 0.51, 0.52, \ldots, 0.9\}$
($p2n100\rho0.5$)	Coefficients and correlation misspecified	β_I and $\rho = -0.2$ or β_{II} and $\rho = -0.2$
($p2n100\rho0.5$)	Error sd and correlation misspecified	$\sigma = 0.4$ and $\rho = -0.2$
($p2n100\rho0.5$)	Expectation of second feature misspecified	$\mu \in \{0.05, 0.1, \ldots, 2, 3\}$
($p2n100\rho0.5$)	Expectation of both features misspecified	$\mu = 10$
($p2n100\rho0.5$)	Variance of second feature misspecified	$\sigma^2 \in \{1.05, 1.1, \ldots, 1.5, 2, 3\}$
($p2n100\rho0.5$)	Variance of both features misspecified	$\sigma^2 = 5$
($p2n100\rho0.5$)	Mean and variance of both features misspecified	$\mu = 10$, $\sigma^2 = 5$
($p2n100\rho0.5$)	Distribution of second feature misspecified as Gaussian mixture with $N(0,10)$	$\alpha \in \{0.01, 0.02, \ldots, 0.05\}$
($p2n100\rho0.5$)	Distribution of second feature misspecified as Gaussian mixture with $N(3,1)$	$\alpha \in \{0.01, 0.02, \ldots, 0.05\}$
($p2n100\rho0.5$)	Distribution of second feature misspecified as log-normal	$logN(0,1)$
($p2n100\rho0.5$)	Distribution of second feature misspecified as Bernoulli	$\pi \in \{0.2, 0.35, 0.4, 0.45, 0.5\}$
($p2n100\rho0.5$)	Error distribution misspecified	$t(10)$, $t(3)$, $\chi^2(1)$, $\chi^2(5)$
($p10n100\rho0.2$)	Error sd misspecified	$\sigma \in \{0.1, 0.2, 0.4, 0.5\}$
($p10n100\rho0.2$)	Correlation misspecified	$\rho \in \{0, 0.1, 0.21, 0.22, \ldots, 0.5, 0.6, \ldots, 0.9\}$, $\rho \in \{(-0.9)^{\|i-j\|}, \ldots, (-0.1)^{\|i-j\|}, 0.1^{\|i-j\|}, 0.2^{\|i-j\|}, 0.21^{\|i-j\|}, \ldots, 0.5^{\|i-j\|}, 0.6^{\|i-j\|}, \ldots, 0.9^{\|i-j\|}\}$ (single block)
($p10n100\rho0.2$)	Coefficients and correlation misspecified	β_I and $\rho = 0.5$
($p10n100\rho0.2$)	Error sd and correlation misspecified	$\sigma = 0.4$ and $\rho = 0.5$
($p10n100\rho0.2$)	Expectation of second half of features misspecified	$\mu \in \{0.05, 0.1, \ldots, 2, \}$
($p10n100\rho0.2$)	Variance of second half of features misspecified	$\sigma^2 \in \{1.05, 1.1, \ldots, 2, 5\}$
($p10n100\rho0.2$)	Distribution of second half of features misspecified as Gaussian mixture with $N(0,10)$	$\alpha \in \{0.01, 0.02, \ldots, 0.05\}$
($p10n100\rho0.2$)	Distribution of second half of features misspecified as Gaussian mixture with $N(3,1)$	$\alpha \in \{0.01, 0.02, \ldots, 0.05\}$
($p10n100\rho0.2$)	Distribution of second half of features misspecified as log-normal	$logN(0,1)$
($p10n100\rho0.2$)	Distribution of second half of features misspecified as Bernoulli	$\pi \in \{0.3, 0.35, 0.4, 0.45, 0.5\}$
($p10n100\rho0.2$)	Error distribution misspecified	$t(10)$, $t(3)$, $\chi^2(1)$, $\chi^2(5)$
($p10n50\rho0.2$)	Error sd misspecified	$\sigma \in \{0.1, 0.2, 0.4, 0.5\}$
($p10n50\rho0.2$)	Correlation misspecified	$\rho \in \{0, 0.1, 0.21, 0.22, \ldots, 0.9\}$, $\rho \in \{(-0.9)^{\|i-j\|}, \ldots, (-0.1)^{\|i-j\|}, 0.1^{\|i-j\|}, 0.2^{\|i-j\|}, 0.21^{\|i-j\|}, \ldots, 0.5^{\|i-j\|}, 0.6^{\|i-j\|}, \ldots, 0.9^{\|i-j\|}\}$ (single block)

Table 1. Complete list of deviations from true scenarios

True scenario	Type of deviation	Values
$(p10n50\rho0.2)$	Coefficients and correlation misspecified	β_I and $\rho = 0.5$
$(p10n50\rho0.2)$	Error sd and correlation misspecified	$\sigma = 0.4$ and $\rho = 0.5$
$(p10n50\rho0.2)$	Expectation of second half of features misspecified	$\mu \in \{0.05, 0.1, \ldots, 3, \}$
$(p10n50\rho0.2)$	Variance of second half of features misspecified	$\sigma^2 \in \{1.05, 1.1, \ldots, 5\}$
$(p10n50\rho0.2)$	Distribution of second half of features misspecified as Gaussian mixture with $N(0, 10)$	$\alpha \in \{0.01, 0.02, \ldots, 0.1\}$
$(p10n50\rho0.2)$	Distribution of second half of features misspecified as Gaussian mixture with $N(3, 1)$	$\alpha \in \{0.01, 0.02, \ldots, 0.1\}$
$(p10n50\rho0.2)$	Distribution of second half of features misspecified as log-normal	$logN(0, 1)$
$(p10n50\rho0.2)$	Distribution of second half of features misspecified as Bernoulli	$\pi \in \{0.3, 0.35, 0.4, 0.45, 0.5\}$
$(p10n50\rho0.2)$	Error distribution misspecified	$t(10)$, $t(3)$, $\chi^2(1)$, $\chi^2(5)$
$(p50n100\rho0.2)$	Error sd misspecified	$\sigma \in \{0.1, 0.2, 0.4, 0.5\}$
$(p50n100\rho0.2)$	Correlation misspecified	$\rho \in \{-0.01, 0, 0.1, 0.21, 0.22, \ldots, 0.9\}$, $\rho \in \{(-0.9)^{\lvert i-j\rvert}, \ldots, (-0.1)^{\lvert i-j\rvert}, 0.1^{\lvert i-j\rvert}, 0.2^{\lvert i-j\rvert}, 0.21^{\lvert i-j\rvert}, \ldots, 0.99^{\lvert i-j\rvert}\}$ (five blocks)
$(p50n100\rho0.2)$	Coefficients and correlation misspecified	β_I and $\rho = 0.5$
$(p50n100\rho0.2)$	Error sd and correlation misspecified	$\sigma = 0.4$ and $\rho = 0.5$
$(p50n100\rho0.2)$	Expectation of second half of features misspecified	$\mu \in \{0.05, 0.1, \ldots, 5\}$
$(p50n100\rho0.2)$	Expectation of all features misspecified	$\mu = 1$
$(p50n100\rho0.2)$	Variance of second half of features misspecified	$\sigma^2 \in \{0.1, 0.11, \ldots, 0.99, 1.05, 1.1, \ldots, 10, 10.1, \ldots, 20, 32, 64, 128, 256, 512, 1024, 2048, 4096, 8192, 16384, 32768, 65536, 131072\}$
$(p50n100\rho0.2)$	Distribution of second half of features misspecified as Gaussian mixture with $N(0, 10)$	$\alpha \in \{0.01, 0.02, \ldots, 0.99\}$
$(p50n100\rho0.2)$	Distribution of second half of features misspecified as Gaussian mixture with $N(3, 1)$	$\alpha \in \{0.01, 0.02, \ldots, 0.99\}$
$(p50n100\rho0.2)$	Distribution of second half of features misspecified as log-normal	$logN(0, 1)$
$(p50n100\rho0.2)$	Distribution of second half of features misspecified as Bernoulli	$\pi \in \{0.3, 0.35, 0.4, 0.45, 0.5\}$
$(p50n100\rho0.2)$	Error distribution misspecified	$t(10)$, $t(3)$, $\chi^2(1)$, $\chi^2(5)$
$(p50n100\rho0.2^{\lvert i-j\rvert})$	Error sd misspecified	$\sigma \in \{0.1, 0.2, 0.4, 0.5\}$
$(p50n100\rho0.2^{\lvert i-j\rvert})$	Correlation misspecified	$\rho \in \{(-0.9)^{\lvert i-j\rvert}, \ldots, (-0.1)^{\lvert i-j\rvert}, 0.1^{\lvert i-j\rvert}, 0.21^{\lvert i-j\rvert}, 0.22^{\lvert i-j\rvert}, \ldots, 0.99^{\lvert i-j\rvert}\}$ (five blocks), $\rho \in \{0, 0.2, 0.21, \ldots, 0.9\}$ (no blocks)
$(p50n100\rho0.2^{\lvert i-j\rvert})$	Coefficients and correlation misspecified	β_I and $\rho = 0$
$(p50n100\rho0.2^{\lvert i-j\rvert})$	Error sd and correlation misspecified	$\sigma = 0.4$ and $\rho = 0$
$(p50n100\rho0.2^{\lvert i-j\rvert})$	Expectation of second half of features misspecified	$\mu \in \{0.05, 0.1, \ldots, 5\}$
$(p50n100\rho0.2^{\lvert i-j\rvert})$	Variance of second half of features misspecified	$\sigma^2 \in \{0.1, 0.11, \ldots, 0.99, 1.05, 1.1, \ldots, 10, 10.1, \ldots, 20\}$
$(p50n100\rho0.2^{\lvert i-j\rvert})$	Distribution of second half of features misspecified as Gaussian mixture with $N(0, 10)$	$\alpha \in \{0.01, 0.02, \ldots, 0.8\}$
$(p50n100\rho0.2^{\lvert i-j\rvert})$	Distribution of second half of features misspecified as Gaussian mixture with $N(3, 1)$	$\alpha \in \{0.01, 0.02, \ldots, 0.8\}$

Table 1. Complete list of deviations from true scenarios

True scenario	Type of deviation	Values
$(p50n100\rho 0.2^{\|i-j\|})$	Distribution of second half of features misspecified as log-normal	$logN(0,1)$
$(p50n100\rho 0.2^{\|i-j\|})$	Distribution of second half of features misspecified as Bernoulli	$\pi \in \{0.3, 0.35, 0.4, 0.45, 0.5\}$
$(p50n100\rho 0.2^{\|i-j\|})$	Error distribution misspecified	$t(10)$, $t(3)$, $\chi^2(1)$, $\chi^2(5)$
$(p50n100\rho 0.5^{\|i-j\|})$	Error sd misspecified	$\sigma \in \{0.1, 0.2, 0.4, 0.5\}$
$(p50n100\rho 0.5^{\|i-j\|})$	Correlation misspecified	$\rho \in \{(-0.9)^{\|i-j\|}, (-0.8)^{\|i-j\|}, \ldots, (-0.1)^{\|i-j\|}, 0.1^{\|i-j\|}, 0.2^{\|i-j\|}, \ldots, 0.4^{\|i-j\|}, 0.51^{\|i-j\|}, 0.52^{\|i-j\|}, \ldots, 0.99^{\|i-j\|}\}$ (five blocks), $\rho \in \{0, 0.1, 0.2, \ldots, 0.4, 0.51, , 0.52 \ldots, 0.9\}$ (no blocks)
$(p50n100\rho 0.5^{\|i-j\|})$	Coefficients and correlation misspecified	β_I and $\rho = 0$
$(p50n100\rho 0.5^{\|i-j\|})$	Error sd and correlation misspecified	$\sigma = 0.4$ and $\rho = 0$
$(p50n100\rho 0.5^{\|i-j\|})$	Expectation of second half of features misspecified	$\mu \in \{0.05, 0.1, \ldots, 5\}$
$(p50n100\rho 0.5^{\|i-j\|})$	Variance of second half of features misspecified	$\sigma^2 \in \{0.1, 0.11, \ldots, 0.99, 1.05, 1.1, \ldots, 10, 10.1, \ldots, 20\}$
$(p50n100\rho 0.5^{\|i-j\|})$	Distribution of second half of features misspecified as Gaussian mixture with $N(0,10)$	$\alpha \in \{0.01, 0.02, \ldots, 0.8\}$
$(p50n100\rho 0.5^{\|i-j\|})$	Distribution of second half of features misspecified as Gaussian mixture with $N(3,1)$	$\alpha \in \{0.01, 0.02, \ldots, 0.8\}$
$(p50n100\rho 0.5^{\|i-j\|})$	Distribution of second half of features misspecified as log-normal	$logN(0,1)$
$(p50n100\rho 0.5^{\|i-j\|})$	Distribution of second half of features misspecified as Bernoulli	$\pi \in \{0.3, 0.35, 0.4, 0.45, 0.5\}$
$(p50n100\rho 0.5^{\|i-j\|})$	Error distribution misspecified	$t(10)$, $t(3)$, $\chi^2(1)$, $\chi^2(5)$
(quake)	Error sd misspecified	$\sigma \in \{0.1, 0.2, 0.4, 0.5\}$
(quake)	Correlation misspecified	$\rho \in \{(-0.9)^{\|i-j\|}, (-0.8)^{\|i-j\|}, \ldots, (-0.1)^{\|i-j\|}, 0.01^{\|i-j\|}, 0.02^{\|i-j\|}, \ldots, 0.4^{\|i-j\|}, 0.5^{\|i-j\|}, \ldots, 0.9^{\|i-j\|}\}$ (one block), $\rho \in \{0, 0.01, 0.02, \ldots, 0.4, 0.5, , 0.6 \ldots, 0.9\}$ (no blocks)
(quake)	Coefficients and correlation misspecified	β_I and $\rho = 0$
(quake)	Error sd and correlation misspecified	$\sigma = 0.4$ and $\rho = 0$
(quake)	Expectation of second half of features misspecified	$\mu \in \{0.05, 0.1, \ldots, 1, 2\}$
(quake)	Variance of second half of features misspecified	$\sigma^2 \in \{0.1, 0.11, \ldots, 0.99, 1.05, 1.1, \ldots, 1.5, 2, 5\}$
(quake)	Distribution of second half of features misspecified as Gaussian mixture with $N(0,10)$	$\alpha \in \{0.01, 0.02, \ldots, 0.05\}$
(quake)	Distribution of second half of features misspecified as Gaussian mixture with $N(3,1)$	$\alpha \in \{0.01, 0.02, \ldots, 0.05\}$
(quake)	Distribution of second half of features misspecified as log-normal	$logN(0,1)$
(quake)	Distribution of second half of features misspecified as Bernoulli	$\pi \in \{0.3, 0.35, 0.4, 0.45, 0.5\}$
(quake)	Error distribution misspecified	$t(10)$, $t(3)$, $\chi^2(1)$, $\chi^2(5)$
(wine_quality), (pol), (Yolanda)	Error sd misspecified	$\sigma \in \{0.1, 0.2, 0.4, 0.5\}$

Table 1. Complete list of deviations from true scenarios

True scenario	Type of deviation	Values
(wine_quality), (pol)	Correlation misspecified	$\rho \in \{(-0.9)^{\|i-j\|}, (-0.8)^{\|i-j\|}, \ldots, 0.9^{\|i-j\|}\}$ (two blocks),<br>$\rho \in \{0, 0.1, 0.2, \ldots, 0.9\}$ (no blocks)
(Yolanda)	Correlation misspecified	$\rho \in \{(-0.9)^{\|i-j\|}, (-0.8)^{\|i-j\|}, \ldots, 0.9^{\|i-j\|}\}$ (ten blocks),<br>$\rho \in \{0, 0.1, 0.2, \ldots, 0.9\}$ (no blocks)
(wine_quality), (pol), (Yolanda)	Coefficients and correlation misspecified	β_I and $\rho = 0$
(wine_quality), (pol), (Yolanda)	Error sd and correlation misspecified	$\sigma = 0.4$ and $\rho = 0$
(wine_quality), (pol), (Yolanda)	Expectation of second half of features misspecified	$\mu \in \{0.05, 0.1, \ldots, 5\}$
(wine_quality), (pol), (Yolanda)	Variance of second half of features misspecified	$\sigma^2 \in \{0.1, 0.11, \ldots, 0.99, 2, 5\}$
(wine_quality), (pol), (Yolanda)	Distribution of second half of features misspecified as Gaussian mixture with $N(0, 10)$	$\alpha \in \{0.01, 0.02, \ldots, 0.99\}$
(wine_quality), (pol), (Yolanda)	Distribution of second half of features misspecified as Gaussian mixture with $N(3, 1)$	$\alpha \in \{0.01, 0.02, \ldots, 0.99\}$
(wine_quality), (pol), (Yolanda)	Distribution of second half of features misspecified as log-normal	$logN(0, 1)$
(wine_quality), (pol), (Yolanda)	Distribution of second half of features misspecified as Bernoulli	$\pi \in \{0.3, 0.35, 0.4, 0.45, 0.5\}$
(wine_quality), (pol), (Yolanda)	Error distribution misspecified	$t(10)$, $t(3)$, $\chi^2(1)$, $\chi^2(5)$
