# Supplementary material for: Simulation study to evaluate when Plasmode simulation is superior to parametric simulation in estimating the mean squared error of the least squares estimator in linear regression
Source: PLoS One. 2024 May 15;19(5):e0299989. doi: 10.1371/journal.pone.0299989 (PMC11095703; doi:10.1371/journal.pone.0299989)
Supplement: S1 Appendix — (PDF) [file pone.0299989.s006.pdf]

S1 Appendix to: When is Plasmode simulation superior to parametric simulation when estimating the MSE of the least squares estimator in linear regression?

Marieke Stolte[1*], Nicholas Schreck[2], Alla Slynko[3], Maral Saadati[2], Axel Benner[2], Jörg Rahnenführer[1], Andrea Bommert[1]

1 Department of Statistics, TU Dortmund University, Dortmund, North Rhine-Westphalia, Germany
2 Division of Biostatistics, German Cancer Research Center, Heidelberg, Baden-Wuerttemberg, Germany
3 Department of Statistics and Actuarial Science, University of Waterloo, Waterloo, Ontario, Canada

Simulation of Bernoulli, log normal, and Gaussian mixture variables with fixed correlations

In each case, we first draw data from a multivariate normal and then transform some of the variables. To get $Ber(\pi)$-distributed data, we dichotomize at the π-quantile u_π of the respective normal distribution, i.e. values of the normal variable tha are smaller than the π-quantile of this normal are set to one, values larger than the quantile are set to zero. To get to log-normal data, we use the exponential function on the respective normal variables. To get to Gaussian mixture variables, we generate two normal variables according to the two distributions to be mixed and then take the observations from the first variable with a probability of α and otherwise the observations of the second variable.

The main challenge is to ensure that the resulting variables fulfill a given covariance structure. For this, we have to find a matching covariance matrix for the underlying multivariate normal variables such that the transformed variables have the given covariance structure. The solutions to this problem for each type of transformation will be presented in the following. Since we want to be able to mix each type of variable with normal variables for our simulation, we need the underlying covariances for pairs of transformed variables as well as for pairs where one variable is transformed while the other variable stays normal. Note that positive definiteness of the desired covariance matrix does not ensure positive definiteness of the underlying covariance matrix. In cases where the determined covariance matrix of the underlying multivariate normal distribution is not positive definite, we still use it for sampling since we checked the distributions of the resulting variables and they looked good even in that case.

Bernoulli

Let $X_1 \sim N(\mu_1, \sigma_1^2)$ a normal variable that will not be transformed, $X_2, X_3 \sim N(0, 1)$ standard normal variables used to generate two Bernoulli variables and denote by $\sigma_{ij} = \mathbb{C}\text{ov}(X_i, X_j)$ the covariances of the original variables. By setting $Y_1 = \mathbb{1}(X_2 \leq u_{\pi_1})$ and $Y_2 = \mathbb{1}(X_3 \leq u_{\pi_2})$ we get $Y_1 \sim Ber(\pi_1)$ and $Y_2 \sim Ber(\pi_2)$ where $\pi_i \in (0, 1),\ i = 1, 2$.

[1] derived the correlation between two Bernoulli variables generated from standard normals as above as

$$\mathbb{C}\mathrm{or}(Y_1, Y_2) = \frac{F_{X_2,X_3}(u_{\pi_1}, u_{\pi_2}) - \pi_1\pi_2}{\sqrt{\pi_1(1-\pi_1)\cdot\pi_2(1-\pi_2)}},$$

where F_{X_2,X_3} denotes the multivariate normal distribution function of X_2 and X_3 that is of $N\left(\begin{pmatrix}0\\0\end{pmatrix}, \begin{pmatrix}1 & \sigma_{23}\\ \sigma_{23} & 1\end{pmatrix}\right)$. This means, that if we want to set the correlation of the transformed variables to ρ, we have to solve

$$F_{X_2,X_3}(u_{\pi_1}, u_{\pi_2}) \overset{!}{=} \rho \cdot \sqrt{\pi_1(1-\pi_1)\cdot\pi_2(1-\pi_2)} + \pi_1\pi_2$$

for σ_{23}. Since there is no closed form for F_{X_2,X_3} we solve the equation numerically by performing a grid search over $\sigma_{23} \in [-1, 1]$ in steps of 10^{-4} and taking the value for which the resulting value of the left-hand side is closest to the required value of the right-hand side.

The correlation between a Bernoulli variable generated from a standard normal and another untransformed normal variable is given as follows.

$$\begin{aligned}
\mathbb{C}\mathrm{ov}(X_1, Y_1) &= \mathbb{C}\mathrm{ov}(X_1, \mathbb{1}(X_2 \le u_{\pi_1}))\\
&= \mathbb{E}(X_1 \cdot \mathbb{1}(X_2 \le u_{\pi_1})) - \mathbb{E}(X_1)\,\mathbb{E}(\mathbb{1}(X_2 \le u_{\pi_1}))\\
&= \int\int_{\mathbb{R}} x_1 \cdot \mathbb{1}(x_2 \le u_{\pi_1}) f_{X_1,X_2}(x_1, x_2)\,\mathrm{d}x_1\,\mathrm{d}x_2 - \mu_1\pi_1\\
&= \int_{-\infty}^{u_{\pi_1}} \int_{\mathbb{R}} x_1 f_{X_1,X_2}(x_1, x_2)\,\mathrm{d}x_1\,\mathrm{d}x_2 - \mu_1\pi_1\\
&= \int_{-\infty}^{u_{\pi_1}} \left[\int_{\mathbb{R}} x_1 f_{X_1|X_2}(x_1|x_2)\,\mathrm{d}x_1\right] f_{X_2}(x_2)\,\mathrm{d}x_2 - \mu_1\pi_1\\
&= \int_{-\infty}^{u_{\pi_1}} \mathbb{E}\left[X_1|X_2 = x_2\right] f_{X_2}(x_2)\,\mathrm{d}x_2 - \mu_1\pi_1\\
&= \int_{-\infty}^{u_{\pi_1}} \left[\mu_1 + \sigma_{12}x_2\right] f_{X_2}(x_2)\,\mathrm{d}x_2 - \mu_1\pi_1\\
&= \int_{-\infty}^{u_{\pi_1}} \mu_1 f_{X_2}(x_2)\,\mathrm{d}x_2 + \int_{-\infty}^{u_{\pi_1}} \left[\sigma_{12}x_2\right] f_{X_2}(x_2)\,\mathrm{d}x_2 - \mu_1\pi_1\\
&= \mu_1\mathbb{P}(X_2 \le u_{\pi_1}) + \sigma_{12} \underbrace{\mathbb{E}(X_2|X_2 \le u_{\pi_1})}_{\text{Expectation of truncated normal}} P(X_2 \le u_{\pi_1}) - \mu_1\pi_1\\
&= \mu_1\pi_1 + \sigma_{12}\left[\mathbb{E}(X_2) - \sigma_2\varphi(u_{\pi_1})/\Phi(u_{\pi_1})\right]\pi_1 - \mu_1\pi_1\\
&= -\sigma_{12}\varphi(u_{\pi_1}),
\end{aligned}$$

where φ and Φ denote the density and cumulative distribution function of a standard normal distribution

$$\Rightarrow \mathbb{C}\mathrm{or}(X_1, Y_1) = -\frac{\sigma_{12}\varphi(u_{\pi_1})}{\sqrt{\sigma_1^2\pi_1(1-\pi_1)}},$$

so we get the solution

$$\sigma_{12} \overset{!}{=} -\frac{\rho\sqrt{\sigma_1^2\pi_1(1-\pi_1)}}{\varphi(u_{\pi_1})}.$$

Log normal

Let $X_i \sim N(\mu_i, \sigma_i^2), i = 1, 2, 3$ normal variable and denote by $\sigma_{ij} = \mathbb{C}\text{ov}(X_i, X_j)$ the covariances of the original variables. By setting $Y_1 = \exp(X_2)$ and $Y_2 = \exp(X_3)$ we get $Y_1 \sim N(\mu_2, \sigma_2^2)$ and $Y_2 \sim N(\mu_3, \sigma_3^2)$.

For the correlations between two transformed log-normal variables we obtain

$$\mathbb{C}\text{or}(Y_1, Y_2) = \frac{\exp(\sigma_{23}) - 1}{\sqrt{(\exp(\sigma_2^2) - 1)(\exp(\sigma_3^2) - 1)}}$$

analogously to the results of [2] for log normals generated from standard normals. Therefore, we have to set

$$\sigma_{23} \stackrel{!}{=} \log\left(\rho\sqrt{(\exp(\sigma_2^2) - 1)(\exp(\sigma_3^2) - 1)} + 1\right).$$

Note, that the lower bound of the possible correlations between Y_1 and Y_2 might be larger than -1 depending on σ_2^2 and σ_3^2, e.g. for standard normals ($\sigma_2^2 = \sigma_3^2$), the lower bound for ρ that can be reached is ≈ -0.632.

The correlation between a log-normal variable generated from a normal and another untransformed normal variable is given as follows.

$$\begin{aligned}
\mathbb{C}\text{ov}(X_1, Y_1) &= \mathbb{C}\text{ov}(X_1, \exp(X_2)) \\
&= \mathbb{E}(X_1 \cdot \exp(X_2)) - \mathbb{E}(X_1)\,\mathbb{E}(\exp(X_2)) \\
&= \mathbb{E}\left[\mathbb{E}\left(X_1 \exp(X_2) | X_2\right)\right] - \mu_1 \exp\left(\mu_2 + \frac{\sigma_2^2}{2}\right) \\
&= \mathbb{E}\left[\exp(X_2)\,\mathbb{E}\left(X_1 | X_2\right)\right] - \mu_1 \exp\left(\mu_2 + \frac{\sigma_2^2}{2}\right) \\
&= \mathbb{E}\left[\exp(X_2)\left(\mu_1 + \sigma_{12}/\sigma_2^2\left(X_2 - \mu_2\right)\right)\right] - \mu_1 \exp\left(\mu_2 + \frac{\sigma_2^2}{2}\right) \\
&= \mathbb{E}\left[\exp(X_2)\mu_1\right] + \sigma_{12}/\sigma_2^2\,\mathbb{E}\left[\exp(X_2)X_2\right] - \sigma_{12}/\sigma_2^2\mu_2\,\mathbb{E}\left[\exp(X_2)\right] \\
&\quad - \mu_1 \exp\left(\mu_2 + \frac{\sigma_2^2}{2}\right) \\
&= \mu_1 \exp\left(\mu_2 + \frac{\sigma_2^2}{2}\right) + \sigma_{12}/\sigma_2^2\,\mathbb{E}\left[\exp(X_2)X_2\right] - \sigma_{12}/\sigma_2^2\mu_2 \exp\left(\mu_2 + \frac{\sigma_2^2}{2}\right) \\
&\quad - \mu_1 \exp\left(\mu_2 + \frac{\sigma_2^2}{2}\right) \\
&= \sigma_{12}/\sigma_2^2 \int_{\mathbb{R}} x_2 \exp x_2 f_{X_2}(x_2)\,\mathrm{d}x_2 - \sigma_{12}/\sigma_2^2\mu_2 \exp\left(\mu_2 + \frac{\sigma_2^2}{2}\right) \\
&= \sigma_{12}/\sigma_2^2 \exp\left(\mu_2 + \frac{\sigma_2^2}{2}\right)(\mu_2 + \sigma_2^2) - \sigma_{12}/\sigma_2^2\mu_2 \exp\left(\mu_2 + \frac{\sigma_2^2}{2}\right) \\
&= \sigma_{12} \exp\left(\mu_2 + \frac{\sigma_2^2}{2}\right) \\
\Rightarrow \mathbb{C}\text{or}(X_1, Y_1) &= \frac{\sigma_{12} \exp\left(\mu_2 + \frac{\sigma_2^2}{2}\right)}{\sqrt{\sigma_1^2 \cdot [\exp(\sigma_2^2) - 1] \exp(2\mu_2 + \sigma^2)}}
\end{aligned}$$

So our solution is given as

$$\sigma_{12} \stackrel{!}{=} \frac{\rho\sqrt{\sigma_1^2 \cdot [\exp(\sigma_2^2) - 1] \exp(2\mu_2 + \sigma^2)}}{\exp\left(\mu_2 + \frac{\sigma_2^2}{2}\right)}$$

Gaussian mixture

Let $X_i \sim N(\mu_i, \sigma_i^2), i = 1, \dots, 5$ normal variable and denote by $\sigma_{ij} = \mathbb{C}\text{ov}(X_i, X_j)$ the covariances of the original variables. Additionally let $\Delta_i \sim Ber(\alpha_i), i = 1, 2$ be independent of all X_i. By setting $Y_1 = \Delta_1 X_2 + (1 - \Delta_1) X_3$ and $Y_2 = \Delta_2 X_4 + (1 - \Delta_2) X_5$ we get $Y_1 \sim \alpha_1 N(\mu_2, \sigma_2^2) + (1 - \alpha_1) N(\mu_3, \sigma_3^2)$ and $Y_2 \sim \alpha_2 N(\mu_4, \sigma_4^2) + (1 - \alpha_2) N(\mu_5, \sigma_5^2)$.

For the transformed variables it holds

$$
\begin{aligned}
\mathbb{E}(Y_1) &= \alpha_1 \mu_2 + (1 - \alpha_1) \mu_3 \\
\mathbb{E}(Y_2) &= \alpha_2 \mu_4 + (1 - \alpha_2) \mu_5 \\
\mathbb{V}\text{ar}(Y_1) &= \alpha_1 \sigma_2^2 + (1 - \alpha_1) \sigma_3^2 + \alpha_1 (1 - \alpha_1^2)(\mu_2 - \mu_3)^2 \\
\mathbb{V}\text{ar}(Y_2) &= \alpha_2 \sigma_4^2 + (1 - \alpha_2) \sigma_5^2 + \alpha_2 (1 - \alpha_2^2)(\mu_4 - \mu_5)^2 \\
\mathbb{E}(Y_1 Y_2) &= \alpha_1 \alpha_2 (\sigma_{24} + \mu_2 \mu_4) + (1 - \alpha_1) \alpha_2 (\sigma_{34} + \mu_3 \mu_4) + \alpha_1 (1 - \alpha_2)(\sigma_{25} + \mu_2 \mu 5) \\
&\quad + (1 - \alpha_1)(1 - \alpha_2)(\sigma_{35} + \mu_3 \mu 5).
\end{aligned}
$$

$$
\Rightarrow \mathbb{C}\text{ov}(Y_1, Y_2) = \alpha_1 \alpha_2 \sigma_{24} + (1 - \alpha_1) \alpha_2 \sigma_{34} + \alpha_1 (1 - \alpha_2) \sigma_{25} + (1 - \alpha_1)(1 - \alpha_2) \sigma_{35}
$$

$$
\Rightarrow \mathbb{C}\text{or}(Y_1, Y_2) = \frac{\alpha_1 \alpha_2 \sigma_{24} + (1-\alpha_1) \alpha_2 \sigma_{34} + \alpha_1 (1-\alpha_2) \sigma_{25} + (1-\alpha_1)(1-\alpha_2) \sigma_{35}}{\sqrt{(\alpha_1 \sigma_2^2 + (1-\alpha_1) \sigma_3^2 + \alpha_1 (1-\alpha_1^2)(\mu_2 - \mu_3)^2)(\alpha_2 \sigma_4^2 + (1-\alpha_2) \sigma_5^2 + \alpha_2 (1-\alpha_2^2)(\mu_4 - \mu_5)^2)}}
$$

The correlation between a Gaussian Mixture variable generated from two normals and another untransformed normal variable is given as follows.

$$
\begin{aligned}
\mathbb{E}(Y_1 X_1) &= \alpha_1 \sigma_{12} + \alpha_1 \mu_1 \mu_2 + (1 - \alpha_1) \sigma_{13} + (1 - \alpha_1) \mu_1 \mu_3 \\
\Rightarrow \mathbb{C}\text{ov}(Y_1, X_1) &= \alpha_1 \sigma_{12} + (1 - \alpha_1) \sigma_{13} \\
\Rightarrow \mathbb{C}\text{or}(Y_1, X_1) &= \frac{\alpha_1 \sigma_{12} + (1 - \alpha_1) \sigma_{13}}{\sqrt{\sigma_1^2 (\alpha_1 \sigma_2^2 + (1 - \alpha_1) \sigma_3^2 + \alpha_1 (1 - \alpha_1^2)(\mu_2 - \mu_3)^2)}}.
\end{aligned}
$$

For the Gaussian mixture variables, there is no unique solution. For symmetry reasons we set

$$
\begin{aligned}
&\sigma_{12} = \sigma_{13} = \rho \sqrt{\sigma_1^2 [\alpha_1 \sigma_2^2 + (1 - \alpha_1) \sigma_3^2 + \alpha_1 (1 - \alpha_1^2)(\mu_2 - \mu_3)^2]} \\
&\sigma_{14} = \sigma_{15} = \rho \sqrt{\sigma_1^2 [\alpha_2 \sigma_4^2 + (1 - \alpha_2) \sigma_5^2 + \alpha_2 (1 - \alpha_2^2)(\mu_4 - \mu_5)^2]} \\
&\sigma_{14} = \sigma_{15} = 0 \\
&\sigma_{24} = \sigma_{35} = \sigma_{34} = \sigma_{25} \\
&\quad = \rho \frac{\sqrt{[\alpha_1 \sigma_2^2 + (1 - \alpha_1) \sigma_3^2 + \alpha_1 (1 - \alpha_1^2)(\mu_2 - \mu_3)^2]\,[\alpha_2 \sigma_4^2 + (1 - \alpha_2) \sigma_5^2 + \alpha_2 (1 - \alpha_2^2)(\mu_4 - \mu_5)^2]}}{\alpha_1 \alpha_2 + (1 - \alpha_1) \alpha_2 + \alpha_1 (1 - \alpha_2) + (1 - \alpha_1)(1 - \alpha_2)}.
\end{aligned}
$$

References

1. Emrich LJ, Piedmonte MR. A Method for Generating High-Dimensional Multivariate Binary Variates. The American Statistician. 1991;45(4):302–304. doi:10.1080/00031305.1991.10475828.

2. Astivia OLO, Zumbo BD. Population models and simulation methods: The case of the Spearman rank correlation. British Journal of Mathematical and Statistical Psychology. 2017;70(3):347–367. doi:10.1111/bmsp.12085.
